# Self-expanding metal stent restenosis in obstructive colon diverticulitis mimicking colon cancer: A case report

**DOI:** 10.1016/j.ijscr.2018.10.073

**Published:** 2018-11-01

**Authors:** Ryo Ohta, Ryota Sakon, Manabu Goto, Yuji Tachimori, Koji Sekikawa

**Affiliations:** Department of Surgery, Institute of Gastroenterology, Kawasaki Saiwai Hospital, Kawasaki, Japan

**Keywords:** Self-expanding metal stent, Benign colorectal obstruction, In-stent restenosis, Case report

## Abstract

**Introduction:**

The self-expanding metal stent (SEMS) has been used in malignant colorectal obstruction as a bridge to surgery or for palliative treatment. We report a case of obstructive descending colon diverticulitis in-stent restenosis, which is difficult to distinguish from colon cancer.

**Presentation of case:**

A 48-year-old man presented with abdominal pain. Computed tomography demonstrated a segment of thickened wall in the descending colon with near-complete obstruction. Colonoscopy revealed stenosis over the entire circumference of the descending colon. Biopsy revealed no malignant findings. SEMS was inserted to improve bowel obstruction. However, bowel obstruction did not improve, and stent-in-stent insertion was performed. Because colon cancer could not be completely excluded, left hemicolectomy with lymph node dissection was performed. The pathological examination revealed colon diverticulitis and no malignant finding.

**Discussion:**

Benign colorectal diseases can present with symptoms similar to those of obstructive colorectal cancer. As a result, it was benign colorectal stenosis, but it was thought that resection should be considered when the malignant lesion is suspected. The use of SEMS in benign colorectal disease has been reported a few. Reinsertion of the stent for restenosis, in bridge to surgery, was also considered useful from the viewpoint of avoiding emergent operation and reducing complications.

**Conclusion:**

For colon stenosis suspected of malignant disease, it was thought that resection by inserting SEMS should be taken into consideration even for benign disease as a result.

## Highlights

• We report a case of obstructive descending colon diverticulitis in-stent restenosis, which is difficult to distinguish from colon cancer.
• SEMS placement for colorectal obstruction is useful method not only malignant tumors, but also benign colorectal diseases.
• There is a thing to keep in mind that colorectal benign disease presents a condition similar to that of obstructive colorectal cancer.

## 1 Introduction

Endoscopic deployment of a self-expanding metal stent (SEMS) can provide temporary relief of acute colorectal obstruction. This procedure has been used in malignant colorectal obstruction as a bridge to surgery or palliative treatment [1]. However, there is limited data about this procedure for benign colorectal obstructions including diverticular disease, postoperative stenosis, and inflammatory bowel disease. Although SEMS may permit bowel preparation through elective decompression and avoidance of stoma creation, it often becomes occluded by tumor ingrowth. In general, stent-in-stent insertion leads to good outcomes in patients with malignant colorectal obstruction [2], but there are few reports with regard to benign colorectal obstruction. Using the SCARE criteria [3], we report our experience with a case of SEMS restenosis in obstructive colon diverticulitis mimicking colon cancer.

## 2 Presentation of case

**Fig. 1:**
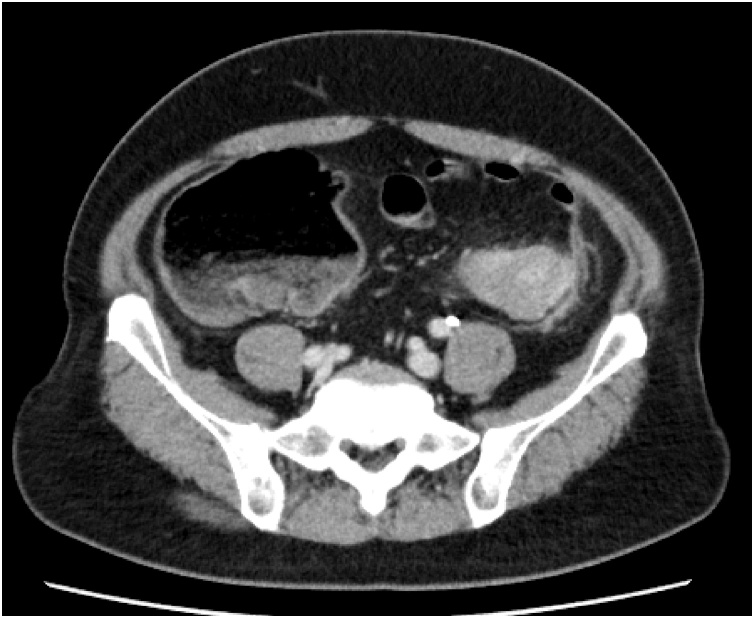
Contrast-enhanced abdominal computed tomography showed a marked thickening and circumferential stenosis in the descending colon.

**Fig. 2:**
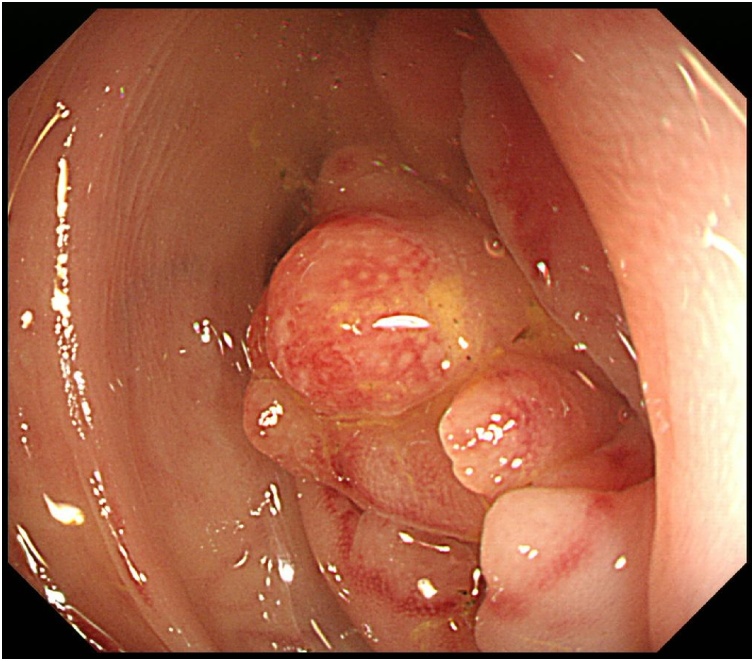
Colonoscopy revealed stenosis over the entire circumference of descending colon.

**Fig. 3:**
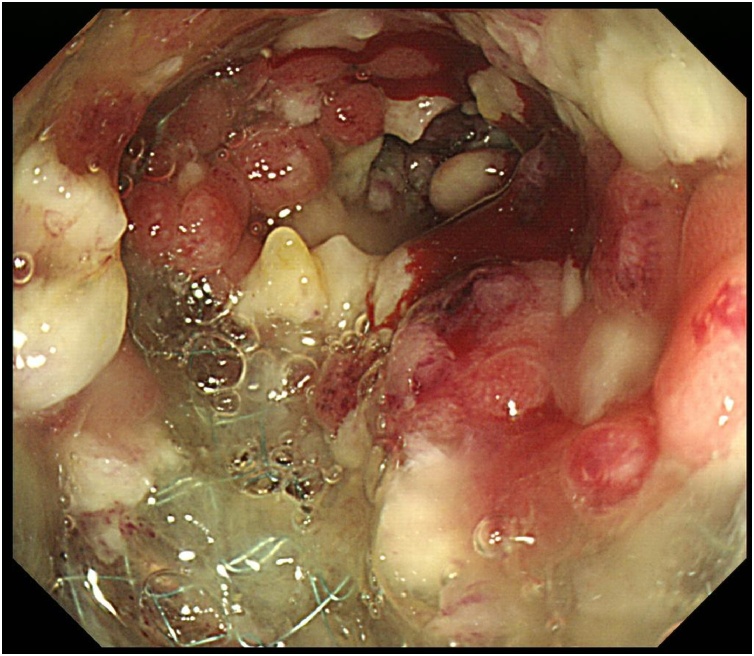
Colonoscopic view of restenosis in the descending colon.

**Fig. 4:**
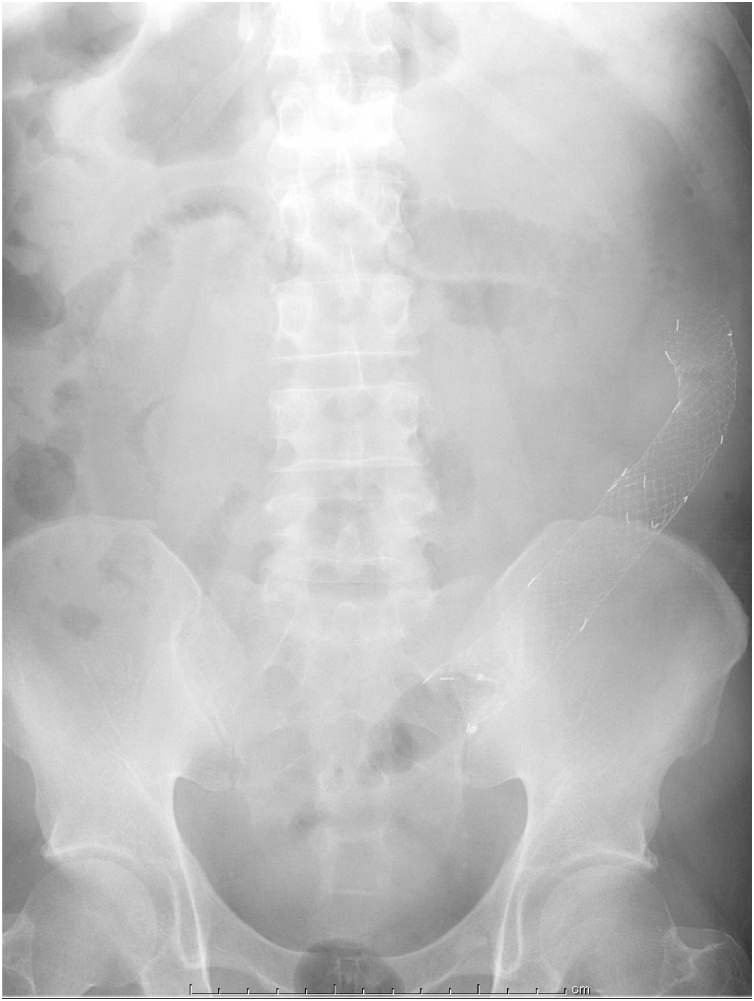
SEMS was additionally inserted to the area of stenosis.

**Fig. 5:**
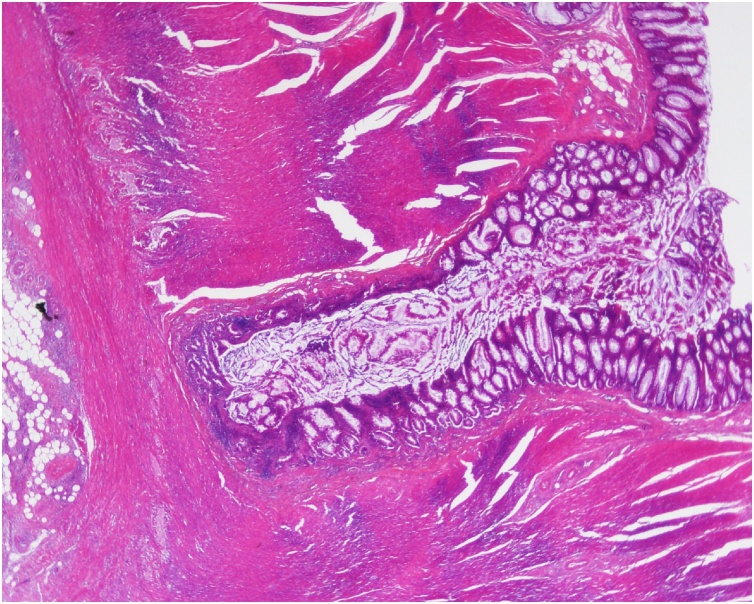
The histological examination of the resected specimen showed diverticulitis with perforation in diverticulosis of the descending colon.

A 48-year-old man complained of abdominal pain, nausea and vomiting. There was no significant past medical history. Diffuse abdominal distension and left abdominal tenderness were found on physical examination. Leukocytosis was detected in laboratory findings. Contrast-enhanced abdominal computed tomography showed marked thickening and circumferential stenosis in the descending colon (Fig. 1). Colonoscopy revealed circumferential stenosis in the entire descending colon (Fig. 2). Biopsy revealed no malignant findings. To improve bowel obstruction, a 22 × 100-mm SEMS (Niti-S, Taewoong Medical, Seoul, Korea) was placed to span the point of obstruction. As bowel obstruction did not improve, a 22 × 80-mm SEMS (Niti-S, Taewoong Medical, Seoul, Korea) was additionally inserted at the area of stenosis (Figs. 3,4). Flatus returned after this procedure. Because descending colon cancer could not be completely excluded, left hemicolectomy with lymph node dissection was performed. During laparotomy, a mass in the descending colon was observed, with adherence of adjacent small intestine. Because invasive descending colon cancer was suspected, partial resection of the small intestine was performed. There were no significant postoperative complications. Histological examination of the resected specimen showed diverticulitis with perforated diverticulosis of the descending colon (Fig. 5). There were no malignant cells.

## 3 Discussion

A transanal decompression tube, emergency colostomy, and Hartmann’s operation have been used for treatment of malignant colorectal obstruction. Since 1991, colorectal stents have been used in the treatment of malignant stenosis [4]. SEMS is used in malignant gastrointestinal obstruction as a bridge to surgery or for palliative treatment. There are many reports of SEMS use in obstructive colorectal cancer, especially as a bridge to one-stage resection or laparoscopic surgery [5]. SEMS use provides preoperative decompression for acute colorectal obstruction, and the possibility of preoperative oral intake and temporary discharge from the hospital. Furthermore, it may be possible to perform an elective operation and avoid stoma creation. This technique is associated with lower morbidity and mortality in malignant colorectal obstruction [6].

Recently SEMS has been used for obstruction or stenosis in benign colorectal disease [7]. However, SEMS use in benign cases is not widely accepted, mainly because of the potential risk of serious adverse events with decreased safety, efficacy, and patency. Compared to use in malignant obstruction, SEMS for benign disease is thought to have higher rates of stent migration, perforation, and restenosis [8]. Therefore, SEMS has been used for benign indications in only 3% of cases [9]. However, these reports demonstrate using SEMS in benign lesions not for bridge to surgery, but for conservative treatment. In our case, we planned SEMS insertion for the purpose of resection rather than conservative treatment. It will be hoped that the results of temporary colon stent placement in colorectal benign disease become clear in the future.

The use of SEMS in the setting of colon cancer is associated with tumor ingrowth rates of 3–46% [10]. On the other hand, little data on stent restenosis in benign disease has been reported. The cause of restenosis is thought to be stent-induced tissue hyperplasia in benign colorectal disease [11]. In our case, bowel obstruction did not improve, suggesting tumor ingrowth, and stent-in-stent insertion was required with the presumptive diagnosis of colon cancer. It is sometimes difficult to differentiate between obstructive colorectal cancer and benign colorectal obstruction. Because colon cancer could not be completely excluded, we performed left hemicolectomy with lymph node dissection. Resection of the lesion even after performing stent in stent is thought to be obtaining a pathological definite diagnosis and avoiding complications due to SEMS insertion. In addition to accumulation of cases, it is hoped that the outcome of stent in stent bridging to surgery to colorectal obstruction becomes clear.

## 4 Conclusion

For colorectal obstruction difficult to distinguish between benign and malignant, it seemed reasonable to resect the lesion even after inserting stent in stent. SEMS bridging to surgery is an effective treatment for any colorectal obstruction and should be introduced positively.

## Conflicts of interest

The authors declare they have no conflict of interests.

## Sources of funding

This study has not received any funding.

## Ethical approval

This case report was approved by the committee of our institute.

## Consent

Written informed consent was obtained from the patient.

## Author contribution

Ryo Ohta – Author, Editing of manuscript.

Ryota Sakon – contributor.

Manabu Goto – contributor.

Yuji Tachimori – contributor.

Koji Sekikawa – contributor.

## Registration of research studies

NA.

## Guarantor

Corresponding author; Ryo Ohta.

## Provenance and peer review

Not commissioned, externally peer reviewed.
